# Patterns of change in high frequency precipitation variability over North America

**DOI:** 10.1038/s41598-017-10827-8

**Published:** 2017-09-18

**Authors:** Susana Roque-Malo, Praveen Kumar

**Affiliations:** 1 Department of Civil and Environmental Engineering, University of Illinois at Urbana Champaign, Urbana, 61801 USA; 2 Department of Atmospheric Sciences, University of Illinois at Urbana Champaign, Urbana, 61801 USA

## Abstract

Precipitation variability encompasses attributes associated with the sequencing and duration of events of the full range of magnitudes. However, climate change studies have largely focused on extreme events. Using analyses of long-term weather station data, we show that high frequency events, such as fraction of wet days in a year and average duration of wet and dry periods, are undergoing significant changes across North America. Further, these changes are more prevalent and larger than those associated with extremes. Such trends also exist for events of a range of magnitudes. Existence of localized clusters with opposing trend to that of broader geographic variation illustrates the role of microclimate and other drivers of trends. Such hitherto unknown patterns over the entire North American continent have the potential to significantly inform our characterization of the resilience and vulnerability of a broad range of ecosystems and agricultural and socio-economic systems. They can also set new benchmarks for climate model assessments.

## Introduction

Variability of high frequency precipitation, that is, the variability associated with non-extreme events such as sequencing and persistence of daily precipitation, plays a significant role in a myriad of terrestrial functions. These include ecosystem and agricultural productivity which are strongly tied to soil-moisture states, biogeochemical processes which are functions of moisture and temperature states, performance of economic systems which depend on sustained availability of water, etc^1^. Although recent research has characterized the non-stationarity of extreme precipitation^2–7^ and its intensification^8–10^ and the change in the mid-range variability such as seasonality^11–15^, little is known about trends of change in sequencing of frequent precipitation events arising from climate change and other anthropogenic impacts.

Understanding and accounting for changes in patterns of high frequency precipitation variability has the potential to inform management and design of myriad systems dependent on hydrologic cycles and to improve predictability of creeping change and associated emergent patterns and risks^16^. For example, agricultural yields and irrigation requirements are affected by changes in daily precipitation and its persistence^17, 18^. If precipitation magnitude is unchanged but precipitation events last longer and are less intense and more frequent, the effect on agricultural management could be different than if precipitation fell in shorter but more intense and infrequent bouts. Similarly, maintenance and continued efficiency of hydropower plants^19^ and other water resources-related systems would be affected by variations in moderate precipitation amounts^20^ potentially to the point of necessitating the revision of structural design standards or water management practices^21, 22^. Aquatic ecosystems are particularly vulnerable to changes in small magnitude events, which can alter natural flow regimes^23^. Therefore, non-stationarity in precipitation sequencing, specifically as it pertains to high frequency precipitation, could prove to be a crucial metric to use to strengthen global climate models’ long-term predictions^24, 25^ and provide much-needed information for a larger network of ecological, social, and economic systems connected by the hydrologic cycle^1^. Unlike extreme precipitation, the cost associated with the repercussions from non-extreme precipitation trends is at present unknown. It may be possible to ascertain a monetary cost of variability in non-extreme precipitation associated with dams and engineering systems, but profound effects on aquatic and terrestrial ecosystems that may not be translatable to direct cost at present could prove to be expensive.

Given the consequential impact of changes in patterns such as the fraction of rainy days in a year and lengths of consecutive wet and dry periods, we investigate the presence and trends of change in such characteristics using long-term data from raingage measurements over North America. Some studies of precipitation persistence conducted in Europe compare seasonal changes in wet and dry periods’ duration and occurrence with cyclone activity and temperature trends^26^ as well as precipitation duration and intensity^27, 28^. One global study of temporal distribution of precipitation emphasizes the importance of light and moderate rainfall events^29^. Our study is the first of its kind to study sequencing pattern changes specifically focusing on the effects on non-extreme precipitation across all of North America, although some precipitation studies have focused on smaller sections of the continent^30–33^ or have focused on extreme precipitation in the United States^34^. Studying the variability at such a large scale enables us to incorporate extensive raingage data (over 3,000 stations) and allows for spatio-temporal analysis on geographic scales ranging from continental to Level III Ecoregions^35^. Two studies, based in the northeastern United States and central United States, studied the changes in distribution of intense precipitation by setting a minimum threshold of the station’s mean precipitation value^32^ and at fixed values corresponding to precipitation ranges defining “moderately heavy”, “very heavy”, and “extreme” precipitation^36^. The northeast US study compared the persistence of wet and dry periods of mean and extreme precipitation in order to compare with changes in total annual precipitation amounts^32^. The study concludes that non-stationarity in precipitation trends is present, noting that both wet persistence and the 95^*th*^ percentile of daily precipitation are increasing. The central United States-based study compares changes in frequency of intense precipitation to several climatological factors, such as tropical cyclone activity and mean annual temperature. Authors show that “very heavy” and “extreme” rain days have become more frequent but that rain event characteristics such as duration and peak hourly rain intensity remain unchanged^36^. Another study based over all of North America analyzes changes in duration of both warm seasons and the dry spells within them at a regional scale by defining minimum daily precipitation thresholds based on both precipitation amount and the corresponding daily temperature^37^. This study demonstrates an increase in persistence of dry periods in eastern and southwestern regions of the United States in the past 40 years.

Drawing on precipitation data from several thousand stations, we choose to translate exceedance or non-exceedance of daily precipitation thresholds to a binary sequence, which allows us to study sequencing patterns, precipitation persistence, and changing annual fractions of days above chosen precipitation thresholds. This method, therefore, includes the important effects of the full range of magnitudes of precipitation and does not exclude the effects of non-extreme, or high frequency, precipitation in the analysis of long-term trends. We are then able to compare independent, long-term trends for both fraction of wet days and persistence of wet and dry periods, and additionally, we can compare those to changes in daily rainfall magnitude. Through this comparison, trends in changes of sequencing in high frequency precipitation events are examined across a large geographic scale.

We explore the hypothesis that average daily precipitation, fraction of days in a year receiving precipitation, and average length of consecutive wet and dry periods, may have independent trends at any station, and the clustering of stations with similar behavior reveal spatially coherent trends. Our findings also lead us to investigate the potential role of regional climate or microclimate as an explanatory variable for changes in local high frequency precipitation patterns.

## Materials and Methods

Daily precipitation data from the Global Historic Climatology Network (GHCN)^38^, one of the most complete global collections of daily precipitation data^39^, for 7,194 stations in North America and Hawaii was made available through Earth Info, Inc. The data set was subjected to numerous, thorough quality control procedures regarding both temperature and precipitation^39, 40^, and has been deemed appropriate for studies that analyze trends in light and heavy precipitation^41, 42^. Data was recorded with a precision of up to a tenth of a millimeter (0.1 *mm*). In the GHCN data set, days with trace amounts of precipitation were flagged and assigned a zero value^38^. Due to the presence of missing or unusable data, daily precipitation records for each station were filtered based on the criteria in Table 1. Stations meeting these requirements numbered 5,259.

**Table 1:** Minimum Requirements for Station Data.

Time Period	Minimum Requirement
Month	≤10 days missing/bad data
Year	≥9 months good data
Decade	≥8 years good data
Total	≥5 consecutive decades

Additionally, a station’s years of coverage were required to not end before the year 2000, which increases the chances stations will overlap in years of coverage so that fair temporal comparisons can be made and with the hope that trends leading up to present-day may aid in future predictions. It is also assumed that “younger” stations would be better maintained and more numerous in general. This additional requirement reduced the number of stations passing the quality control criteria (Table 1) to 3,030.

**Figure 1:**
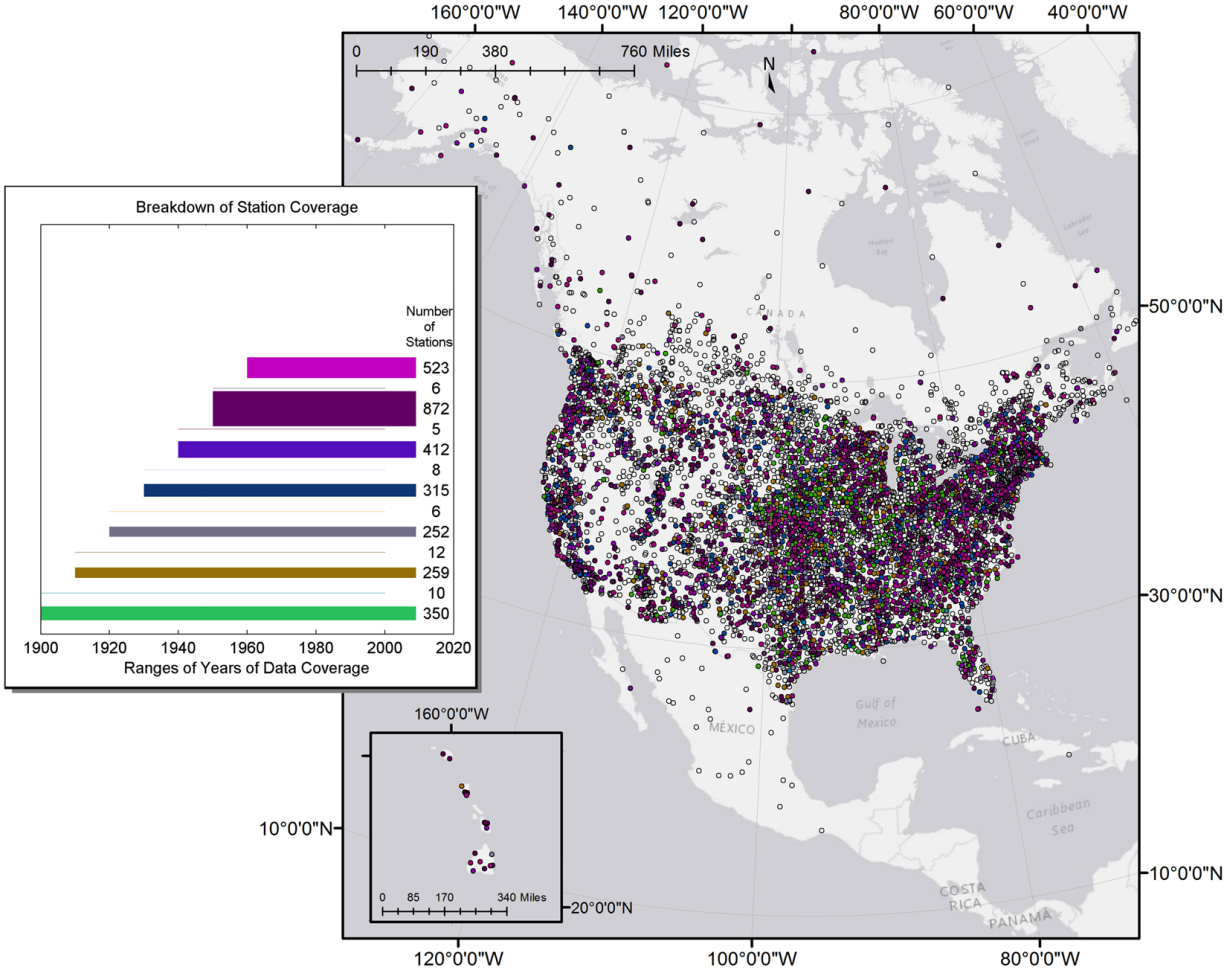
Locations of North American rain gauge stations. Stations passing the quality control criteria listed in Table 1 are color-coded based on the years of coverage as indicated in the inset. Most stations whose data coverage begins in 1920 or earlier are located in the Midwest, while more recent stations appear to be evenly distributed. The majority (60%) of stations were established in 1940 or later. Empty circles indicate stations whose data failed to meet quality control criteria and are excluded from the analyses. In the inset, the bar beginning in 1900 represents stations whose data start in 1900 or earlier, as there are a small number of stations passing quality control criteria whose data start between 1880 and 1900. Also, the number listed to the right of each bar indicates the number of stations with data available for the time period with those particular start and end years. In the analysis, stations with data ending in 2000 or 2010 are combined based on start year because both sets meet criteria to be representative of that particular period (see Materials and Methods). [Map produced with software ArcMap v. 10.4.1, http://desktop.arcgis.com/en/arcmap/].

Of the stations that did not pass filtering, 21% were eliminated outright because the total number of available years (usable or not) was less than the minimum requirement of five decades. Several stations fell into clusters in remote locations, such as the plains areas in southern Saskatchewan, Canada, which suggests the possibility that lack of ease of accessibility contributed to instrument errors or calibration issues, causing breaks in data recording. In addition, stations in Canada have undergone changes in observational practices independent of those in the United States and Mexico, and such station inhomogeneity could introduce bias in data records. However, the number of stations that pass the filtering tests in Canada are only a small fraction of the total number of stations analyzed. The influence of other possible sources of inhomogeneity in station data across all of North America, such as changes in time of observation, were thoroughly considered and do not diminish the robustness of this study. The locations of passing and failing stations are shown in Fig. 1, where filled circles indicate stations whose data passed all analysis requirements, and empty circles indicate failing stations. After filtering, the years of coverage for passing stations fell between 1880 and 2010. The Fig. also indicates the number of stations with different lengths of coverage.

## Results and Discussion

### Temporal and Spatial Patterns of Change

**Figure 2:**
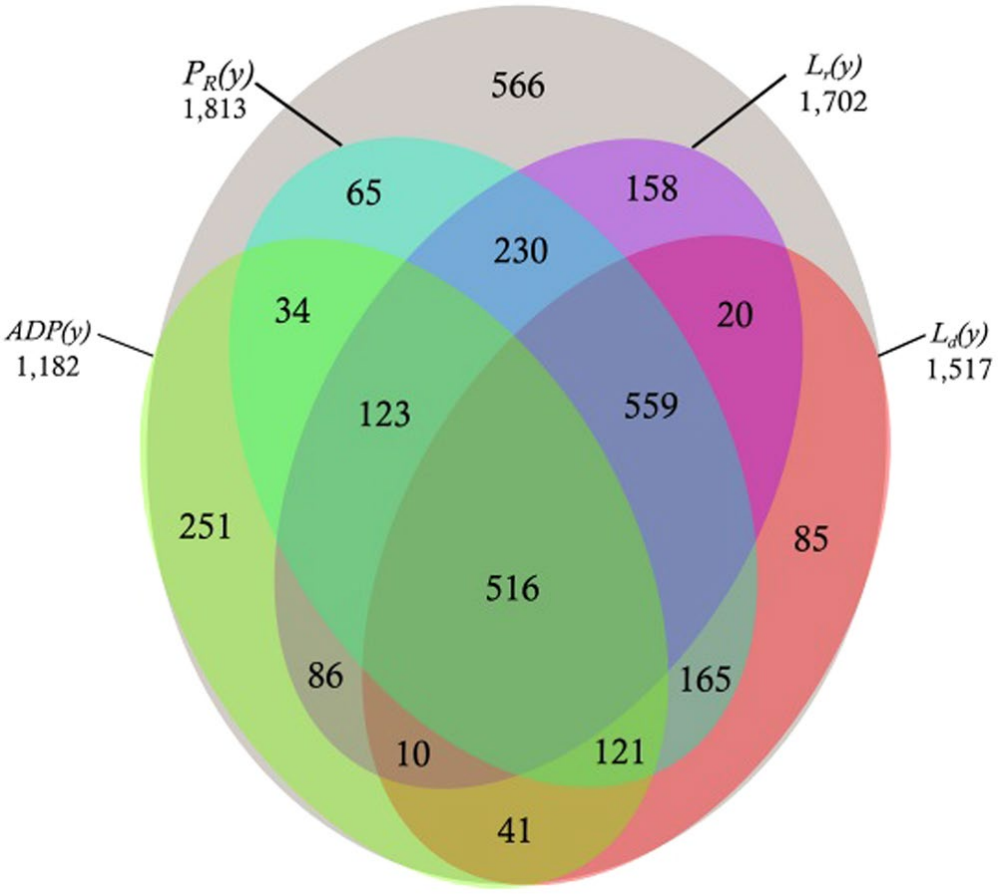
Illustration of number of stations that show trends in different attributes characterizing precipitation variability: average daily precipitation (*ADP(y)*), fraction of wet days in a year (*P*_*R*_(*y*)), and average length of consecutive wet (*L*_*r*_(*y*)) and dry (*L*_*d*_(*y*)) days. These attributes describe different facets of precipitation variability and the results indicate that they can behave independent of one another. The grey area represents 566 stations showing no trend in any of the attributes.

Data from the 3,030 stations that pass this quality control are used to compute the average daily precipitation, *ADP*(*y*), for each year *y* of acceptable data record based on all days in which a measurable amount of rainfall is recorded. Therefore, *ADP*(*y*) is can be equivalently converted to annual total precipitation. Further, the daily precipitation data are converted to a binary sequence based on each day’s exceedance of a daily minimum threshold, *δ*. For each station, the binary sequence *α*_*δ*_(*i*) is defined as:

1 $${\alpha }_{\delta }(i)=\{\begin{array}{c}1\,{\rm{if}}\,R(i) > \delta \\ 0\,{\rm{otherwise}},\end{array}$$

where *R*(*i*) is the recorded precipitation total on the *i* ^th^ day. We define *α*_*δ*_(*i*) as the precipitation/no-precipitation sequence based on each day’s exceedance or non-exceedance of 0.3 *mm*. The lowest recorded daily rainfall amount across all stations was 0.3 *mm*. Therefore, this was chosen as the lowest threshold. However, we also consider higher thresholds associated with the 50^th^, 75^th^, 90^th^ and 95^th^ percentile of nonzero recorded precipitation for each station over its entire period of record (illustrated in Figure S1). These binary sequences of precipitation disregard magnitude but capture the sequencing and persistence of events above a threshold *δ*. By choosing a range of *δ* values, this method enable us to analyze the trends therein, if one exists. Furthermore, from {*α*_*δ*_(*i*)}_*i*_, for each year *y* we compute the fraction of wet days (*P*_*R*_(*y*)), and average length of consecutive wet (*L*_*r*_(*y*)) and dry period (*L*_*d*_(*y*)). The Mann-Kendall test at 5% significance level is used to determine the existence of statistically significant monotonically increasing or decreasing long-term trends for a variable^43^. This test, which is consistent with other studies^29, 32^ is chosen because of its ability to handle gaps in data records^44^, which can be an issue with other statistical tests such as the Sen Slope Method^45^. Slopes (Δ) for trends in each attribute *ADP(y)*, *P*_*R*_(*y*), *L*_*r*_(*y*), and *L*_*d*_(*y*) are determined by linear regression over the entire study period for stations showing statistically significant trends (for illustration, see Figure S2). This approach provides a first order analysis, and there is a possibility that higher order trends or short-term periodic behavior overlain on long-term trends are present at some stations. However, by testing for a monotonically increasing or decreasing behavior over a sufficiently long study period, the overall, long-term trend can be captured. The breakdown of the number of stations showing trends in different attributes is illustrated in Fig. 2. We see that many stations show a statistically significant trend in only one of the attributes and several stations show trends in a combination of these attributes. Finally, 516 stations show trends in all the attributes. These attributes represent different facets of precipitation variability, and the results show that they can act independent of one another, meaning that presence of a trend in one does not mandate that a similar behavior is present in another. Figure S3 in Supplemental Materials compares the distribution of analyzed statistics for all stations across all years of data for both stations passing quality control for trend analysis and for stations in which a statistically significant trend is found.

**Figure 3:**
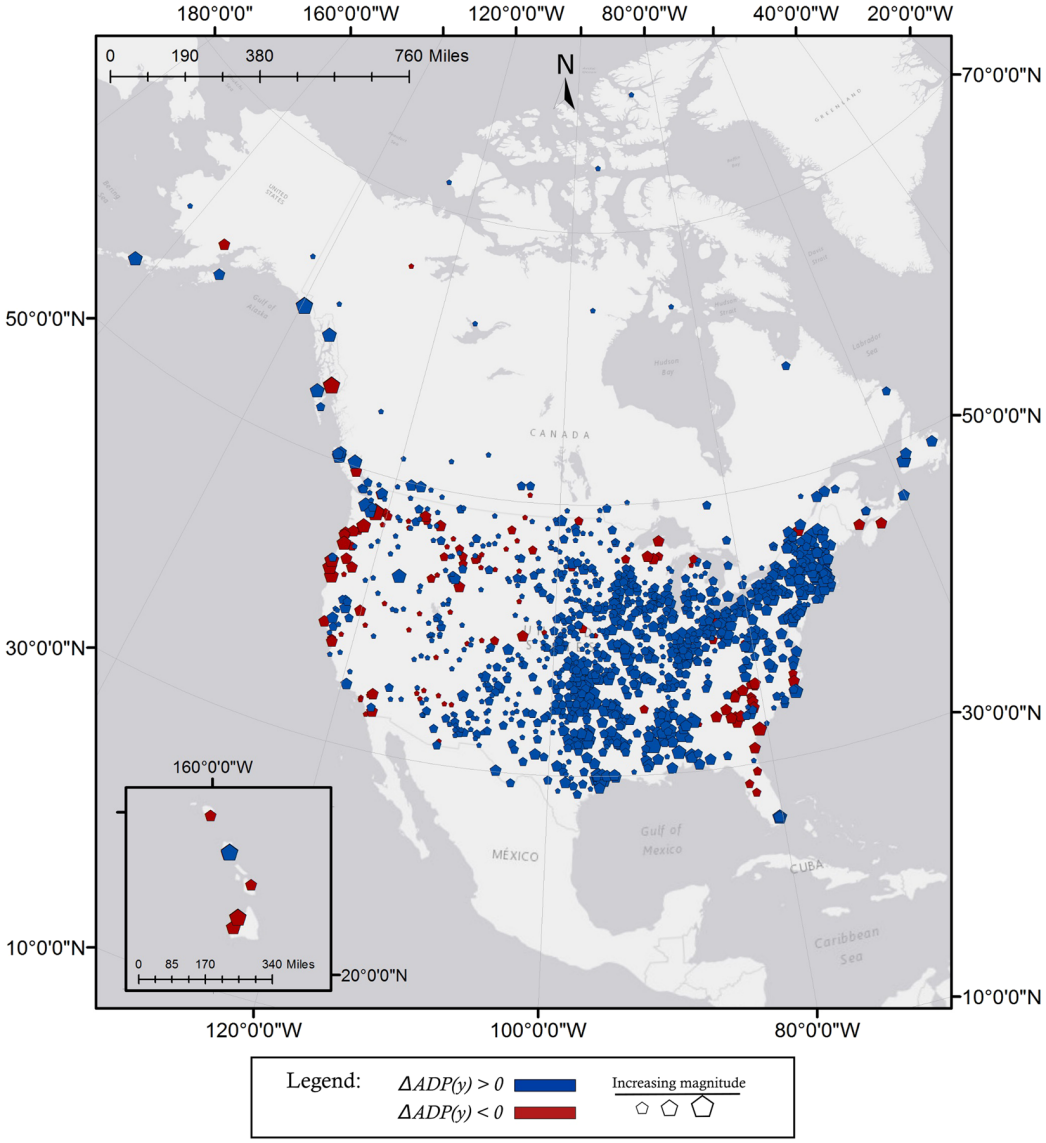
Statistically significant trends in average daily precipitation (*ADP(y)*) were detected in 1,182 stations. Stations that indicated decreasing *ADP(y)* (red symbols) numbered 136 and are mostly located in the Southeast, Pacific Northwest, and several other small pockets. Positive trends in *ADP(y)* (blue symbols) appeared in 1,046 stations located in the U.S. Midwest, South, and Northeast. Symbol size is proportional to trend magnitude. Overall, stations east of the 100^*th*^ meridian west showed the strongest increases in average daily precipitation. [Map produced with software ArcMap v. 10.4.1, http://desktop.arcgis.com/en/arcmap/].

In addition to testing for statistically significant trends in *P*_*R*_(*y*), *L*_*r*_(*y*), and *L*_*d*_(*y*), we tested for the presence of trends in average daily precipitation in a year, *ADP(y)*. In total, 1,182 stations showed a statistically significant trend in *ADP(y)*. The majority of stations that are located in the American Northeast/Canadian Southeast and in the middle of the continent around the Mississippi River indicate positive slopes in *ADP(y)* (Fig. 3). Stations showing decreasing *ADP(y)* were mostly limited to the American Southeast, the Pacific Northwest, and several pockets in the American Southwest and at northern latitudes. These continent-scale patterns are quite similar to those shown previously^46^, although the previous results were based on decadal averages compared to our study’s annual averages. The distribution of slopes, associated with linear trends of change, in *ADP(y)* is presented in Table 2 as Δ*ADP(y)* and in Fig. 6. The median of slope, Δ*ADP(y)*, across all stations with a statistically significant trend is 5.34 × 10^−3^ *mm*/*day*/*yr*, which translates to an additional 97.5 *mm* of precipitation in a given year compared to 50 years prior.

**Table 2:** Distribution of Slopes Associated with Statistically Significant Trends for *δ* = 0.3 *mm*.

*Percentile*	Δ*ADP(y)* (*mm*/*y*)	Δ*PR*(*y*)	Δ*L*_*r*_(*y*) (*days*/*y*)	Δ*L*_*d*_ (*days*/*y*)
1	−1.63 × 10^−2^	−2.22 × 10^−3^	−1.18 × 10^−2^	−1.10 × 10^−1^
5	−5.23 × 10^−3^	−1.21 × 10^−3^	−5.69 × 10^−3^	−5.48 × 10^−2^
10	−1.73 × 10^−3^	−8.31 × 10^−4^	−3.77 × 10^−3^	−3.75 × 10^−2^
25	3.13 × 10^−3^	−1.96 × 10^−4^	1.10 × 10^−3^	−2.15 × 10^−2^
50	5.34 × 10^−3^	5.98 × 10^−4^	2.77 × 10^−3^	−1.13 × 10^−2^
75	8.37 × 10^−3^	9.91 × 10^−4^	4.86 × 10^−3^	−2.96 × 10^−3^
90	1.20 × 10^−2^	1.55 × 10^−3^	7.67 × 10^−3^	2.06 × 10^−2^
95	1.47 × 10^−2^	1.91 × 10^−3^	1.05 × 10^−2^	3.47 × 10^−2^
99	2.14 × 10^−2^	3.11 × 10^−3^	1.88 × 10^−2^	8.02 × 10^−2^

**Figure 4:**
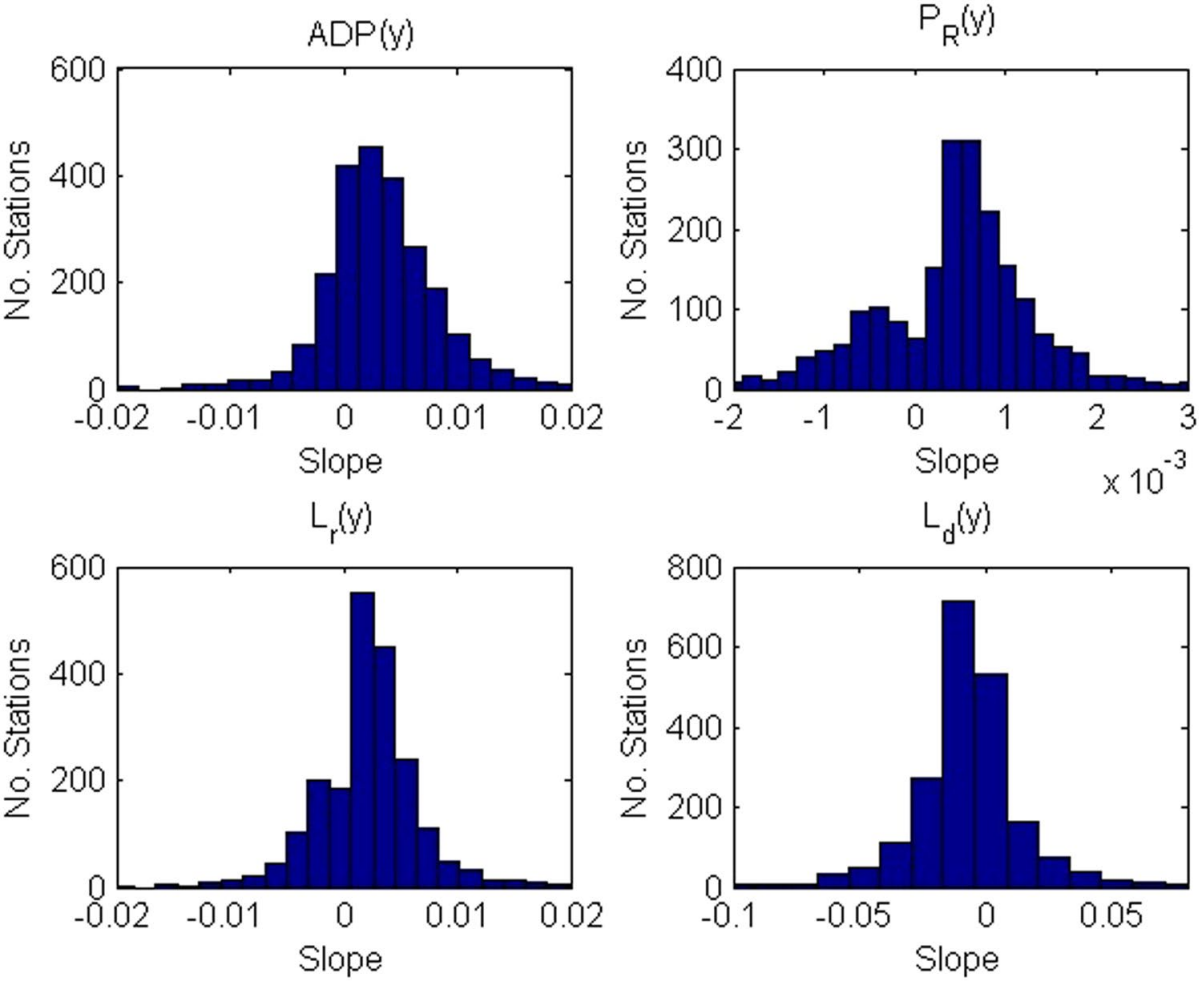
Distribution of Slopes Associated with Statistically Significant Trends for *δ* = 0.3 *mm* for *ADP(y)*, *P*_*R*_(*y*), *L*_*r*_(*y*), and *L*_*d*_(*y*).

From Table 2 (graphically represented in Fig. 4), we also see that across North America, the median Δ*P*_*R*_(*y*), i.e. the median slope of *P*_*R*_(*y*), indicates that the chance of any given day receiving precipitation in exceedance of 0.3 *mm* is increasing by 5.98 × 10^−4^ per year. This means that in 2009, there were an extra 11 days with recorded precipitation compared to 50 years earlier when the median number of days with precipitation was 96 days per year. The median value of Δ*L*_*r*_(*y*), i.e. the median slope of the average number of consecutive days with precipitation, 2.77 × 10^−3^, indicates increasing persistence of precipitation events. The median value for Δ*L*_*d*_ *(y)*, i.e. the median slope of average number of consecutive days without precipitation, is −1.13 × 10^−2^. These results, which include only the stations that show statistically significant trend in the corresponding variable, suggest that for a typical station among those showing a trend in 2009 saw wet periods 0.14 days longer than were seen 50 years earlier (when the median length of consecutive wet days was 1.78 days), and consecutive dry periods were typically half a day shorter than a 50 years earlier (when the median length of consecutive dry days was 4.8 days). Although this combination of trends is expected to be more prevalent, it is not necessarily the rule. It is possible that an area may see more days of precipitation (Δ*P*_*R*_(*y*) > 0), fewer consecutive days of precipitation (Δ*L*_*r*_(*y*) < 0), and shorter periods without rain (Δ*L*_*d*_ *(y*) < 0). This could mean that the area is receiving the same amount of precipitation at smaller, more staggered intervals throughout the year or season, or it could mean that there are more frequent, more intense bouts of rain spread throughout the year depending on Δ*ADP(y)*.

There was not a significant difference in trends seen between the time periods with different start years (Figure S4). However, in order to ensure that stations with different periods of temporal coverage did not introduce a bias, the above analysis was repeated for the same data subjected to the additional requirement that all usable data fall between 1960 and 2009. The resulting median values for Δ*ADP(y)*, Δ*P*_*R*_(*y*), Δ*L*_*r*_(*y*), and Δ*L*_*d*_(*y*) were 4.30 × 10^−3^, 5.03 × 10^−4^, 2.70 × 10^−3^, and −3.10 × 10^−3^, respectively. Because these trends calculated in data from the same temporal periods were consistent with those found using all available usable data spanning different time periods, the latter was chosen to establish long-term trends and ensure that all available data was used to inform on the analysis and inference. This additional testing also allowed for the comparison of trends in the same stations but with different start times (e.g. trends in stations with data coverage from 1920 to 2009 could be compared to trends in those same stations from 1960 to 2009). Again, results from this comparison showed consistency in trends, which lends itself to dispelling the possibility that outliers at the beginning of data sequences influence linear regression results.

**Figure 5:**
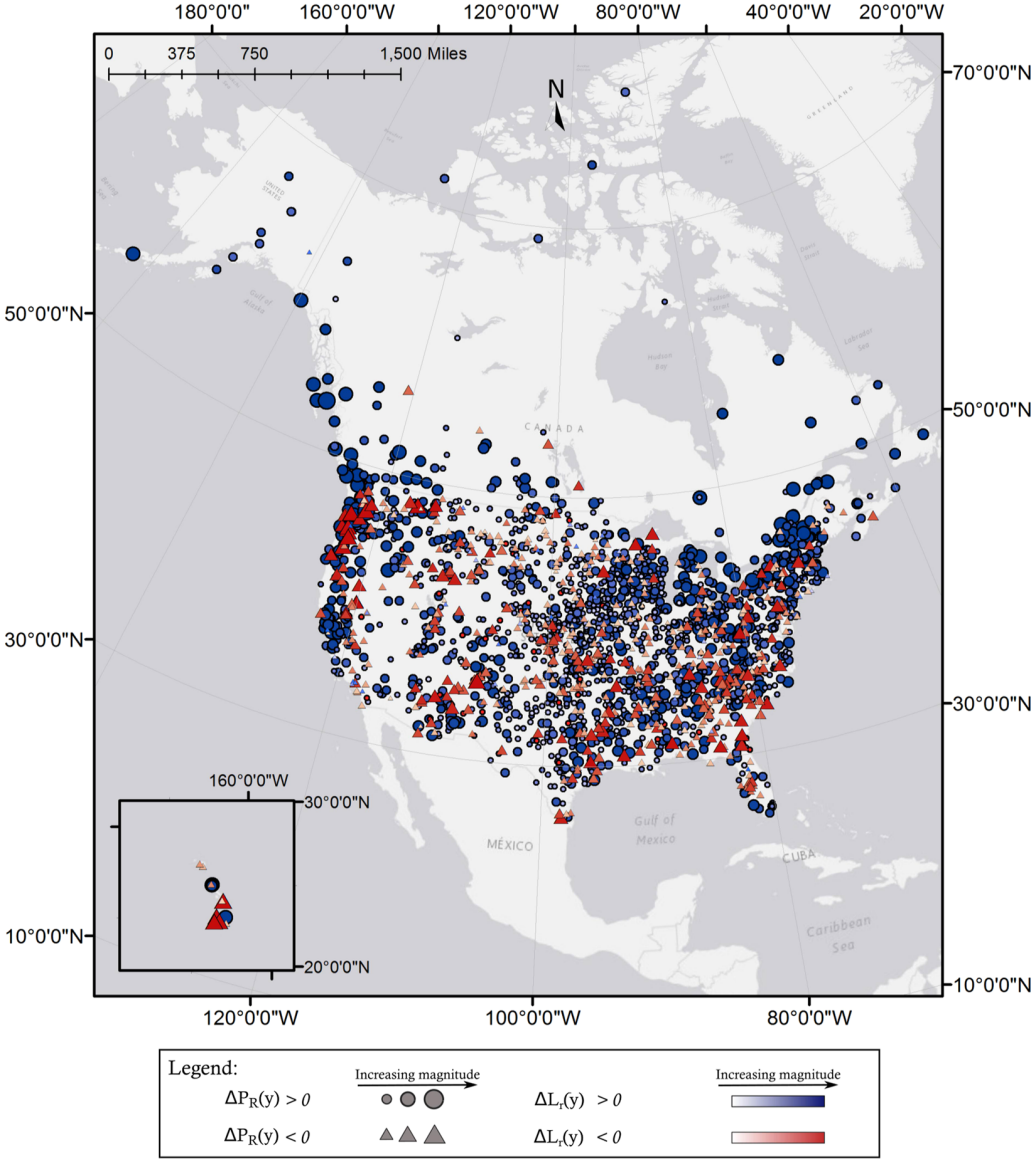
Illustration of the spatial distribution in precipitation variability and length of consecutive wet days. Stations where fraction, *P*_*R*_(*y*), of wet days in a year *y* is increasing are marked with a circle, and stations where it is decreasing are marked with a triangle. The size of the symbol is proportional to the magnitude of the trend. Blue symbols indicate stations where length of consecutive wet days, *L*_*r*_(*y*), is increasing, while red symbols indicate stations where it is decreasing. The color saturation is proportional to the magnitude of the trend. For example, larger deep blue circles indicate stations where both fraction of wet days and length of consecutive wet days in a year are increasing. Similarly, larger deep red triangles indicates stations with decreasing fraction of wet days and decreasing length of wet days in a year. The largest increases in *P*_*R*_(*y*) are found at higher latitudes on the Pacific Northwestern Coast, where they are often accompanied by larger increases in *L*_*r*_(*y*), and in Northeastern states. Inland on the Pacific Coast, in the Southeast, and in some pockets in the Midwest, stations show stronger decreases in *P*_*R*_(*y*). [Map produced with software ArcMap v. 10.4.1, http://desktop.arcgis.com/en/arcmap/].

**Figure 6:**
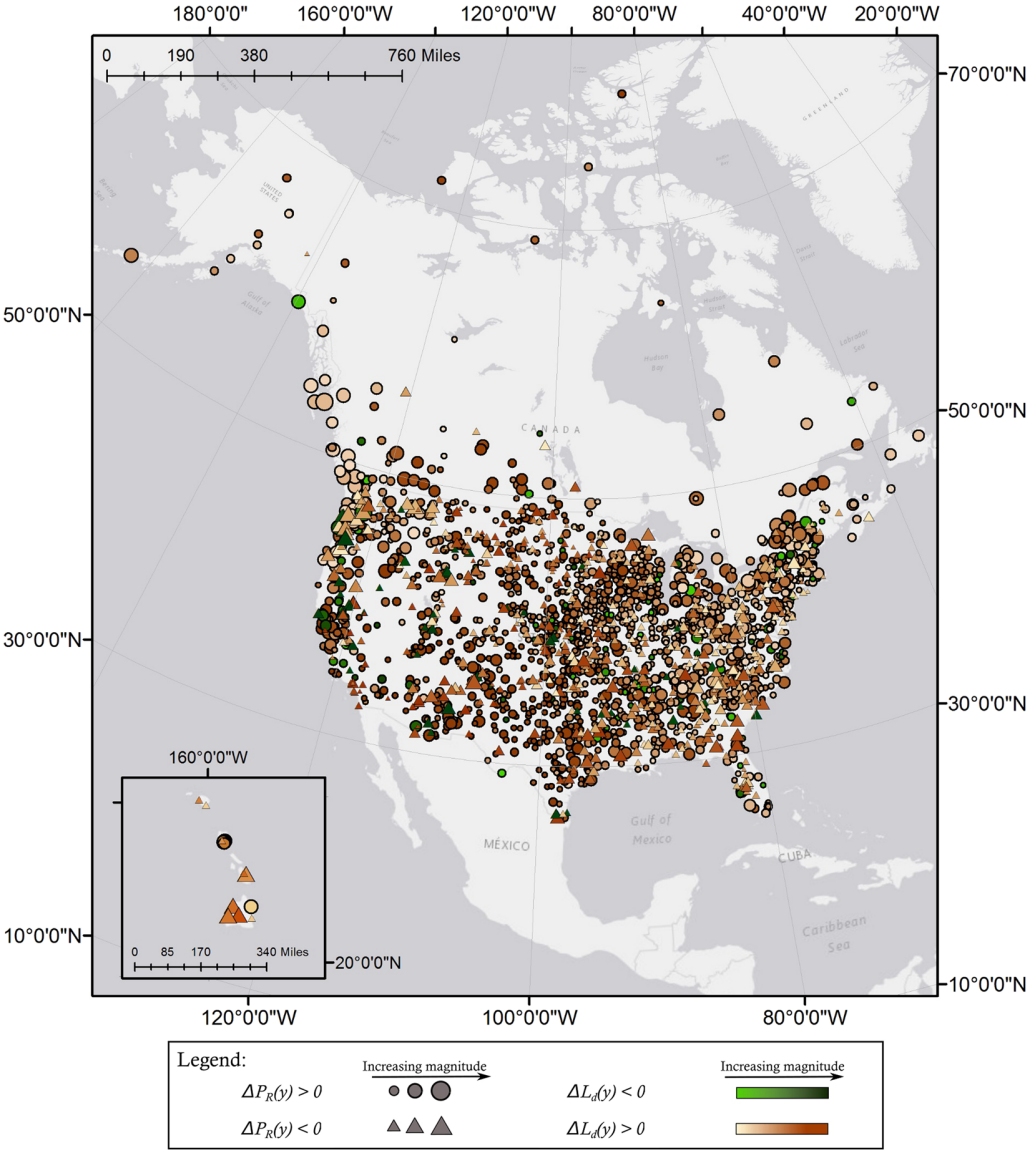
Illustration of the spatial distribution in precipitation variability. Stations where fraction of wet days in a year *y*, *P*_*R*_(*y*), is increasing are marked with a circle, and stations where it is decreasing are marked with a triangle. The size of the symbol is proportional to the magnitude of the trend. Green symbols indicate stations where length of consecutive dry days, *L*_*d*_(*y*), is decreasing, while orange symbols indicate stations where it is increasing. The color saturation is proportional to the magnitude of the trend. For example, larger deep green circles indicate stations where both fraction of wet days is increasing and length of consecutive dry days in a year is decreasing. Similarly, larger deep orange triangles indicate stations with decreasing fraction of wet days and increasing length of dry days in a year. In contrast to Fig. 5, coastal zones do not show such strong trends in *L*_*d*_(*y*). Instead, areas in the Interior Plains, parts of the Rockies, and the Southwest show the strongest changes in *L*_*d*_(*y*). [Map produced with software ArcMap v. 10.4.1, http://desktop.arcgis.com/en/arcmap/].

Figure 5 illustrates the spatial variation of these temporal trends. Nonuniform spatial variation of sequencing patterns is apparent. The Northeastern United States and Pacific Northwest show higher magnitude increase in slopes of both *P*_*R*_(*y*) and *L*_*r*_(*y*). It is observed that higher latitudes across the continent appear to show larger magnitude increases in *P*_*R*_(*y*). Pockets in the northern Appalachian mountains, Gulf Coast states, and part of Hawaii also show strongly positive trends in *P*_*R*_(*y*). The middle of the continent shows increasing slope in both *P*_*R*_(*y*) and *L*_*r*_(*y*), but the magnitudes are relatively smaller. North-central regions of the continent and parts of the American Southeast also appear to show small-magnitude decreases in the slope of *P*_*R*_(*y*) and *L*_*r*_(*y*). However, in several areas dominated by positive Δ*P*_*R*_(*y*), such as the Pacific Northwest and the Northeastern United States, stations showing negative Δ*P*_*R*_(*y*) in large magnitudes are also present. In contrast to the spatial patterns seen in Fig. 5, Fig. 6 shows that trends for change in *L*_*d*_(*y*) are more concentrated to the Interior Plains, parts of the Rockies, and the Southwest. Coastal regions show smaller magnitude trends in *L*_*d*_(*y*) compared to *L*_*r*_(*y*).

Studying precipitation sequencing adds a new dimension to our understanding of changing precipitation patterns, especially in comparisons between precipitation timing and, for example, trends in magnitude. Evidence of this is found in a notable difference between Fig. 3 and Fig. 5. Figure 3 illustrates that the middle of the continent, specifically in the area of Texas, Oklahoma, and Missouri, indicates strongly positive Δ*ADP(y)* over time. However, Fig. 5 illustrates that stations in these regions show lower rates of change in both *P*_*R*_(*y*) and *L*_*r*_(*y*). On the other hand, areas such as the American Northeast/Canadian Southeast overlap with largely positive trends in both *P*_*R*_(*y*), *L*_*r*_(*y*), and *ADP(y)*.

Given the overall positive trends in both Δ*P*_*R*_(*y*) and Δ*ADP(y)*, it may be tempting to generalize that stations with increasing numbers of rainy days per year (i.e., Δ*P*_*R*_(*y*) > 0) would see an increase in magnitude of daily precipitation (i.e., Δ*ADP(y)* > 0), and similarly, that fewer days of precipitation coincide with less recorded daily precipitation. The former is not a poor generalization given that 84% of stations showing positive Δ*P*_*R*_(*y*) indicated increasing trends in daily precipitation amounts. However, of the 527 stations with negative Δ*P*_*R*_(*y*), 62% indicated increasing *ADP(y)*. In total, nearly a third of all stations showing a positive or negative trend in *P*_*R*_(*y*) do not fit the generalization that more rainy days bring more rain or fewer rainy days bring less rain. This result challenges the generally accepted idea that with climate change ‘wet areas get more wet and dry areas get drier’ and that rain events will become more intense^10^, and it is consistent with literature that has identified limitations of this assumption^47^. Depending on the combination of trends in *P*_*R*_(*y*), *L*_*r*_(*y*), and *ADP* for a given station, this generally accepted idea could prove to be an oversimplification. For example, positive Δ*P*_*R*_(*y*) and positive Δ*L*_*r*_(*y*) can coincide with negative Δ*ADP(y)* as shown in Figure S2(d); this combination of trends connotes more dissipated, drawn out precipitation. This finding demonstrates that long-term trends in precipitation patterns cannot be assumed based on knowledge of changes in magnitude or other precipitation metrics.

Seasonal scale analysis of these patterns (Figure S5) reveals intra-annual patterns that may not be visible in overall annual results. Although some regions, such as the American Northeast/Canadian Southeast, appear to undergo fairly consistent changes year-round, the American Southwest, which does not demonstrate particularly clear trends in Fig. 5, shows more strongly seasonal trends, shown in Figure S5(b). In Spring and Autumn, this region shows mostly negative Δ*L*_*r*_(*y*) in combination with both increasing and decreasing *P*_*R*_(*y*). However, in Summer, which coincides with the region’s monsoon season, the region shows an increasing fraction of days with precipitation and longer wet periods; this could have implications for monsoon patterns and many ecological functions closely tied to pulsing precipitation patterns. Figure S5(b) demonstrates localized seasonal patterns, as well. The Central Valley region of California, which shows positive Δ*P*_*R*_(*y*) and Δ*L*_*r*_(*y*) year-round is surrounded by stations indicating negative Δ*L*_*r*_(*y*); this pattern is especially clear in Autumn.

**Figure 7:**
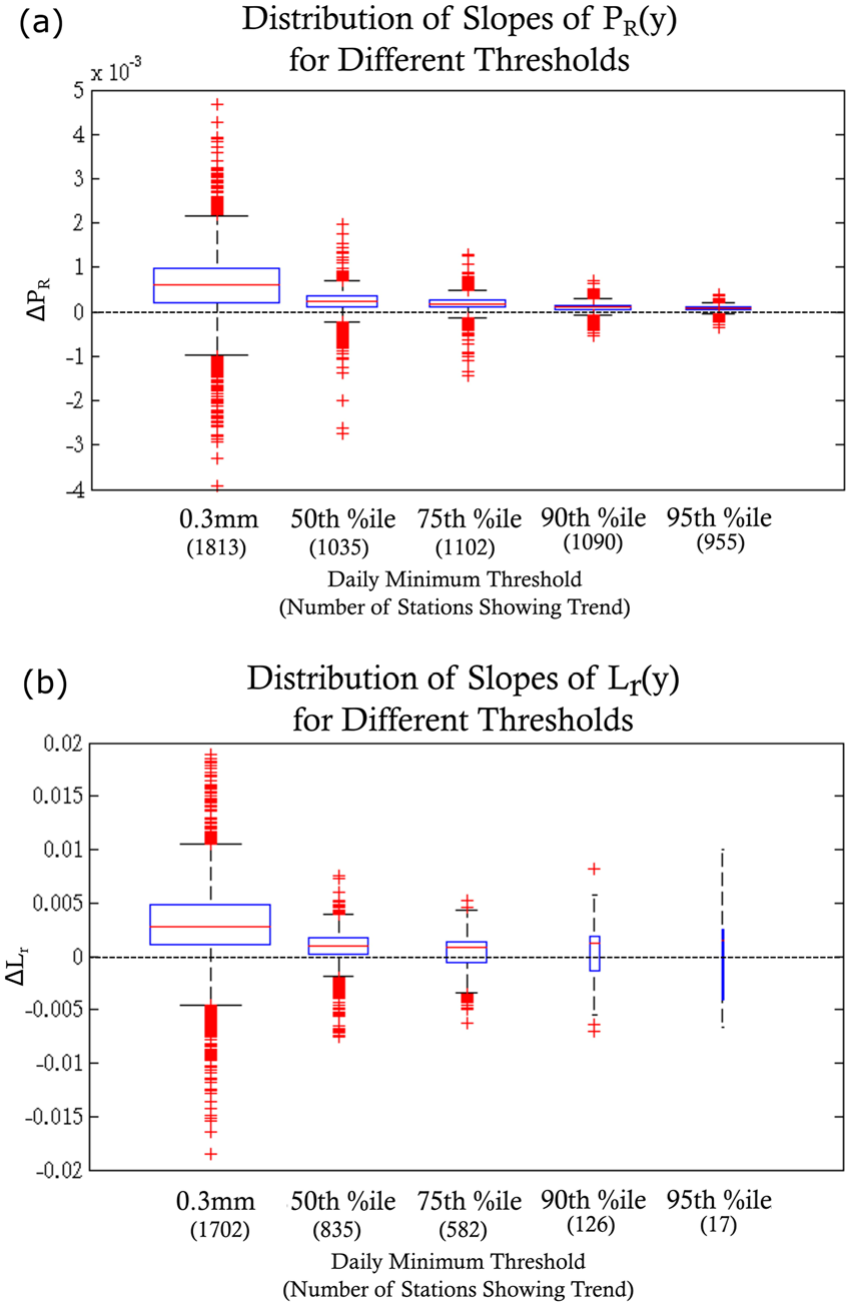
Comparison of the distribution of statistically significant non-zero slopes of *P*_*R*_(*y*) (**a**) and *L*_*r*_(*y*) (**b**). Boxplot widths are proportional to the number of stations showing a trend for each threshold, which are shown in parenthesis below the threshold. The median and range of slopes and the number of stations showing a trend decrease substantially as the daily minimum threshold increases.

Figure 7 summarizes the trend statistics when *δ* is increased to the 50^*th*^, 75^*th*^, 90^*th*^, and 95^*th*^ percentile of precipitation distribution of each station. In addition to the significant decrease in the number of stations showing nonstationarity in precipitation sequencing statistics, the range of Δ*P*_*R*_(*y*) for extreme precipitation also decreases as the threshold increases (Fig. 7). This means that not only is the median Δ*P*_*R*_(*y*) for high frequency precipitation much higher than for low frequency precipitation amounts, but the largest magnitude slopes of high frequency precipitation are much larger than those of low frequency precipitation. These results demonstrate that more stations are experiencing changes in non-extreme precipitation than extreme precipitation.

### Regional Climate or Microclimate Impacts

**Figure 8:**
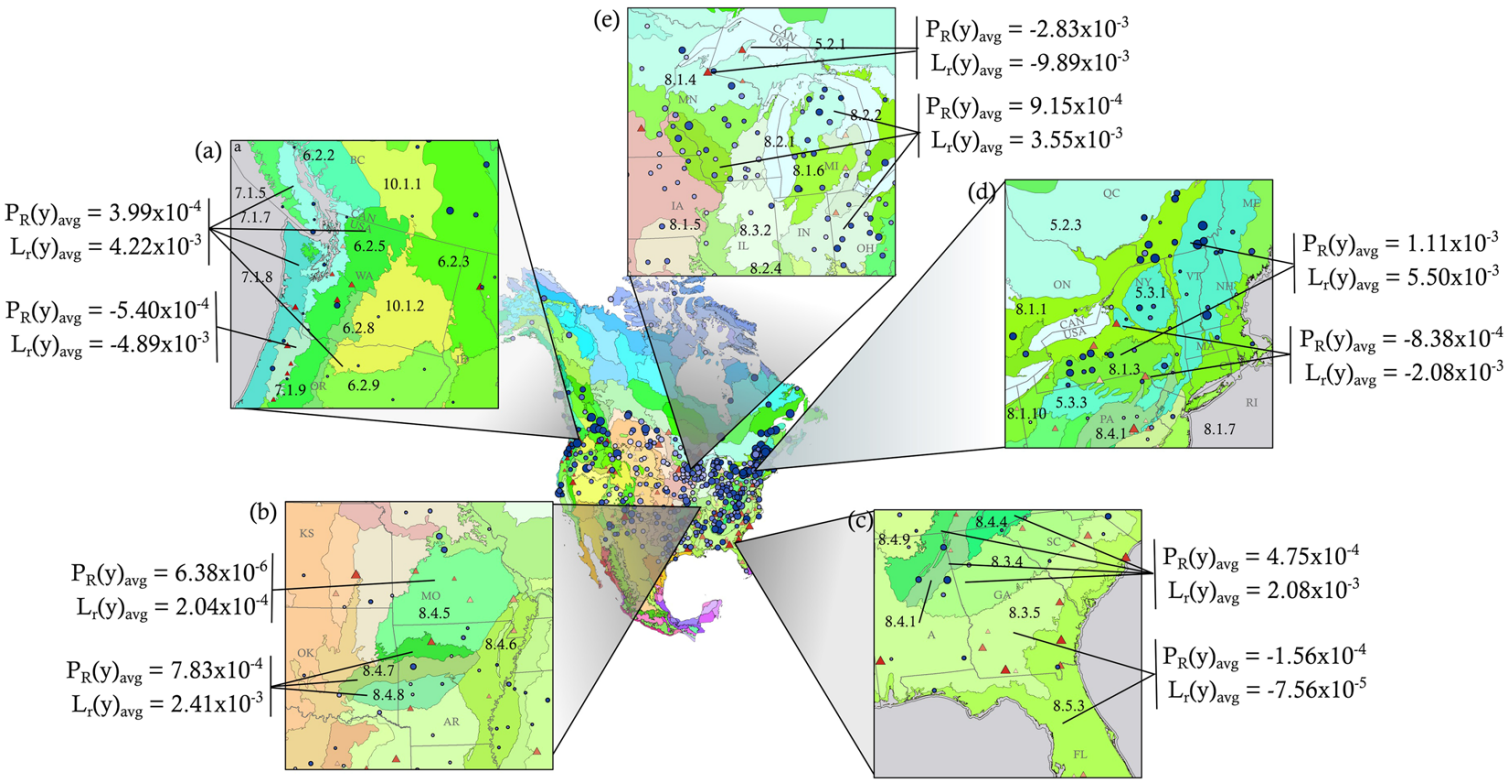
Details of areas exhibiting ecoregional- or topography-based trends in *P*_*R*_(*y*) and *L*_*r*_(*y*). For clarity, only stations appearing in the top quartile (all have Δ*P*_*R*_(*y*) > 0) and bottom quartile (all have Δ*P*_*R*_(*y*) < 0) are shown. Level III North American ecoregions (abbrev. *III-Ecoregion Number*) are color blocked, and specific ecoregions are identified by the number^35^. Regional- or ecoregion-based averages are displayed next to each subfigure, depending on the area of interest described in the text. (**a**) Pacific Northwest, specifically Willamette Valley (*III-7.1.9*). (**b**) Western Ozarks (*III-8.4.5*). (**c**) Southeastern United States, specifically the Southeastern Plains (*III-8.3.5*) and Southern Coastal Plains (*III-8.5.3*) ecoregions. (**d**) American Northeast/Canadian Southeast. (**e**) Great Lakes and surrounding areas. [Map produced with software ArcMap v. 10.4.1, http://desktop.arcgis.com/en/arcmap/].

Our study also shows the spatial clustering of many stations showing opposing trends in *P*_*R*_(*y*), *L*_*r*_(*y*), and *ADP(y)*. Figure 8 highlights five regions that are notable because they consistently show high levels of nonstationarity for both *P*_*R*_(*y*) and *L*_*r*_(*y*) and illustrate the presence of opposing trends in closely located stations. This suggests the hypothesis that regional climate or microclimate variability is responsible for such opposing trends. The spatial anomalies shown in each section in Fig. 8 are explored by analyzing ecoregion characteristics^35^ and changes in land cover as surrogates for microclimatic variations.

Figure 8(a), which highlights the Pacific Northwestern Coast of the United States, suggests that stations in Oregon showing strongly decreasing rates of *P*_*R*_(*y*) and *L*_*r*_(*y*) are mostly located in the Willamette Valley (Level III Ecoregion 7.1.9, or *III-7.1.9*). In contrast, stations showing strongly positive Δ*P*_*R*_(*y*) and Δ*L*_*r*_(*y*) surround the Willamette Valley. The North Cascades ecoregion (*III-6.2.5*) typically receives between 762 and 3810 *mm* of rainfall per year^48^. The immediately adjacent Willamette Valley usually receives between 940 and 1500 *mm* of rainfall per year^49^. Daily precipitation amounts in Willamette Valley have been steadily declining (Figs 3 and S6), which is not the case in areas directly to the north and east. The Willamette Valley differs in seasonal trends, as well. Most stations in the Pacific Northwest demonstrate strongly increasing *P*_*R*_(*y*) and *L*_*r*_(*y*) in every season. However, the Willamette Valley shows that the ecoregion is experiencing decreases in *P*_*R*_(*y*) and *L*_*r*_(*y*) during Autumn and into Winter, times which nearly span rainy season (Figure S5(a)).

Microclimates on a much smaller scale could also be responsible for some long-term trends observed. The American Northeast and Canadian Southeast, shown in Fig. 8(d), consistently shows strongly increasing trends in *P*_*R*_(*y*) and *L*_*r*_(*y*) year-round for all *δ* thresholds except for several stations located in valleys and urban areas. Figure 3 indicates that nearly the entire region is experiencing greater magnitudes of daily precipitation.

In addition to the trends associated with microclimatic gradients across ecoregions, hot spots of opposing trends in precipitation sequencing appear in areas that experience significant anthropogenic changes. The Ozark Highlands ecoregion (*III-8.4.5* in Fig. 8(b)) shows decreasing *P*_*R*_(*y*) and *L*_*r*_(*y*), while neighboring areas indicate the opposite trend. Decreasing trends in the Ozark Highlands are noted for all seasons but Autumn and were confirmed for extreme precipitation, as well. The ecoregions located directly to the south, the Boston Mountains (*III-8.4.6*), Arkansas Valley (*III-8.4.7*), and the Ouachita Mountains (*III- 8.4.8*) show fairly strong increasing *P*_*R*_(*y*) and *L*_*r*_(*y*) trends. Although the Ozark Highlands do receive slightly less average annual precipitation and significantly less annual precipitation in dry years than the surrounding ecoregions^50^, the ecoregion has experienced notably higher rates of growth both in terms of urbanization and transition from natural ecosystem to agriculture^50^.

A similar situation is observed in the Southern Coastal Plains (*III-8.5.3*) and the Southeastern Plains (*III-8.3.5*) ecoregions (Fig. 8(c)). Decreasing trends in *P*_*R*_(*y*) and *L*_*r*_(*y*) are apparent throughout the Southern Coastal Plains ecoregion in Florida, Georgia, and South Carolina, as well as the southeastern foothills of the Appalachians for both non-extreme and extreme (50^*th*^ and 75^*th*^ percentile thresholds) precipitation. Similar to the Ozark Highlands, the Southern Coastal Plains and Southeastern Plains ecoregions have experienced significant land cover change, the highest and second-highest percentage change in the entire Southeastern United States, respectively^51^. Both ecoregions have experienced significant increases in population over time, as well as frequent rotation between natural state, agricultural land, and forested land. The simultaneous influence on precipitation from both macroclimate and anthropogenic factors is discussed in^52^ on a larger scale, but our results suggest that the influence of external forcings can be observed at a smaller, local microclimate scale.

Although the Ozark Highlands, Southern Coastal Plains, and Southeastern Plains ecoregions demonstrate similar sequencing trends, the regions differ greatly when compared in terms of magnitude of precipitation. In Fig. 3, the Ozark Highlands area indicates increasing *ADP(y)*, while the southeastern United States shows strongly negative trends in *ADP(y)*. Similar opposing patterns can be seen in Fig. 8(e), which highlights the ecoregions around the Great Lakes. In general, most stations in this area show Δ*P*_*R*_(*y*) > 0 and Δ*L*_*r*_(*y*) > 0 and also indicate Δ*ADP(y)* > 0; the same is true for negative slopes. However, some stations showed significantly decreasing trends in *ADP(y)* (see Fig. 3). These differences support the contention that precipitation sequencing patterns capture variability that cannot be discerned directly from precipitation amounts.

These observations suggest the possibility that different vegetative covers and topographies could play a more prominent role than previously understood in long-term precipitation sequencing patterns and emphasize that large-scale regions cannot be generalized in climate predictions given the evident strength of some local climates. Consideration of long-term sequencing trends on a local or regional scale suggests that long-term trends in precipitation take on a more complex form, one that is likely determined by a combination of interconnected local climate and anthropogenic factors. The observations presented in this analysis of more nuanced variability in trend highlight the importance of incorporating high frequency precipitation variability as a performance metrics for climate models. However, further study is necessary to characterize the cause of these local trends.

## Electronic supplementary material

Supplementary Information
