# Supplementary material for: Patterns of change in high frequency precipitation variability over North America
Source: Sci Rep. 2017 Sep 18;7:10853. doi: 10.1038/s41598-017-10827-8 (PMC5603571; doi:10.1038/s41598-017-10827-8)
Supplement: Supplementary file 1 — Supplementary Information [file 41598_2017_10827_MOESM1_ESM.pdf]

Patterns of change in high frequency precipitation variability over North America

Susana Roque-Malo and Praveen Kumar

Supplementary Information

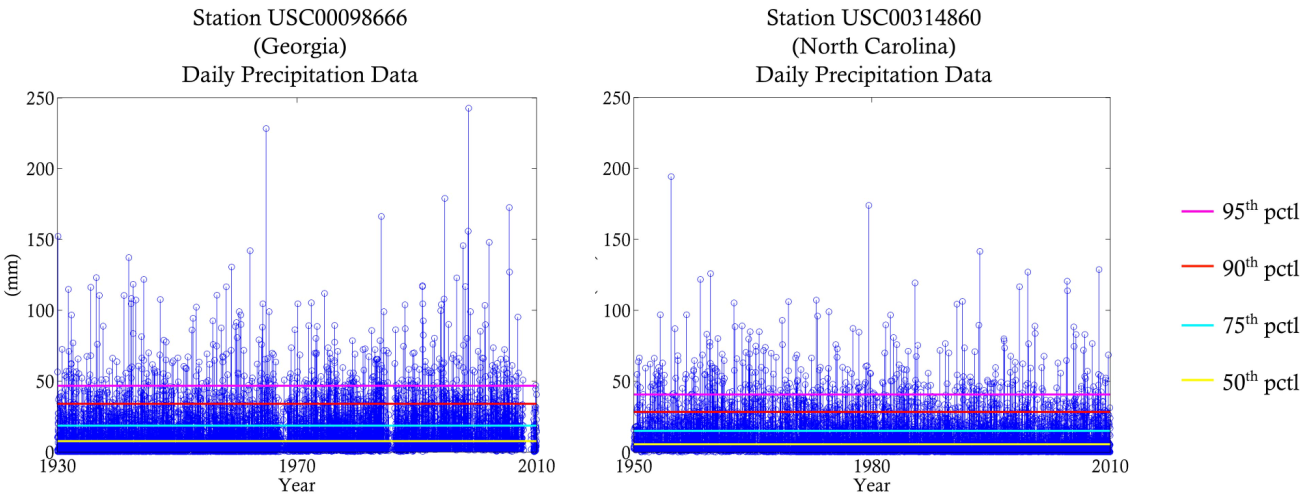

Figure S1. Illustration of different daily minimum thresholds (δ) imposed for low-frequency rainfall analysis. Example stations (station ID shown) cover different years and have different rainfall amounts. Percentiles are calculated based on non-zero daily recorded rainfall amounts for each station.

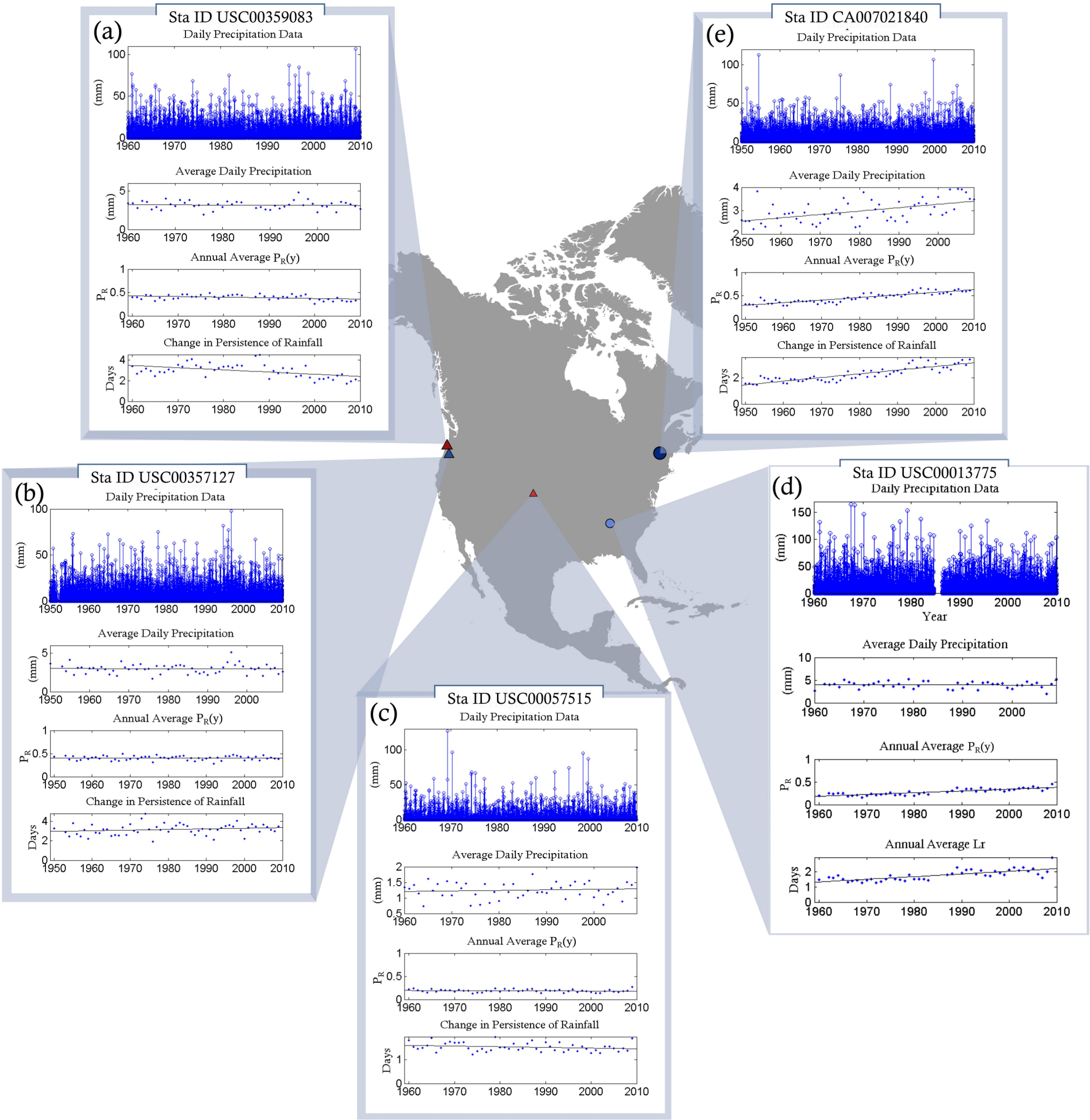

Figure S2. Illustration of different sequencing indicators for five example stations (station IDs shown). (a) $\Delta P_R(y) < 0$, $\Delta L_r(y) < 0$ and $\Delta ADP(y) < 0$. (b) $\Delta P_R(y) < 0$, $\Delta L_r(y) > 0$ and $\Delta ADP(y) < 0$. (c) $\Delta P_R(y) < 0$, $\Delta L_r(y) < 0$ and $\Delta ADP(y) > 0$. (d) $\Delta P_R(y) > 0$, $\Delta L_r(y) > 0$ and $\Delta ADP(y) < 0$. (e) $\Delta P_R(y) > 0$, $\Delta L_r(y) > 0$ and $\Delta ADP(y) > 0$. Several other combinations of trends in slope are possible, but not shown. Symbols on map are simply representative; they are enlarged for clarity and not to scale. [Map produced with software ArcMap v. 10.4.1, http://desktop.arcgis.com/en/arcmap/.]

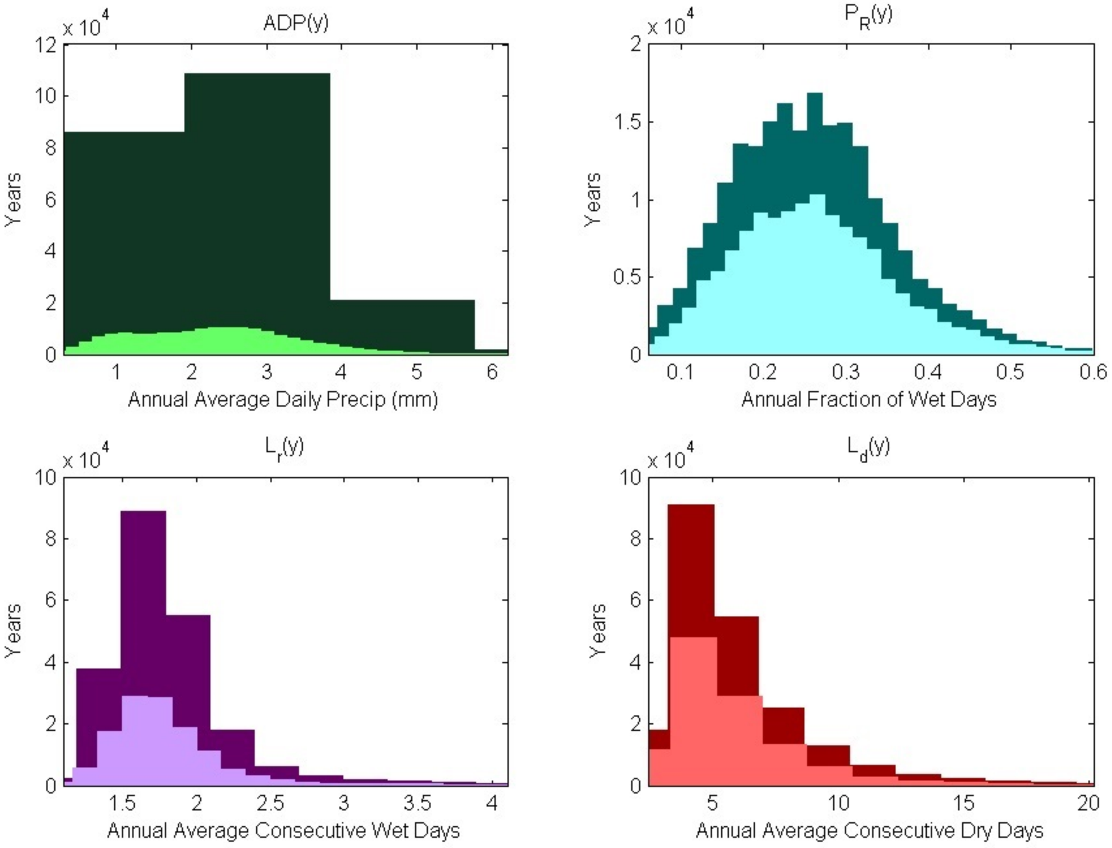

Figure S3. Distribution of analyzed statistics for all stations across all years of data. Histograms with darker shades represent all possible data from stations that pass quality control for trend analysis. Overlain on these are lighter-shaded histograms which represent data from stations in which a statistically significant trend is present. The pictorial display shows data between the 1st and 99th percentile for each variable.

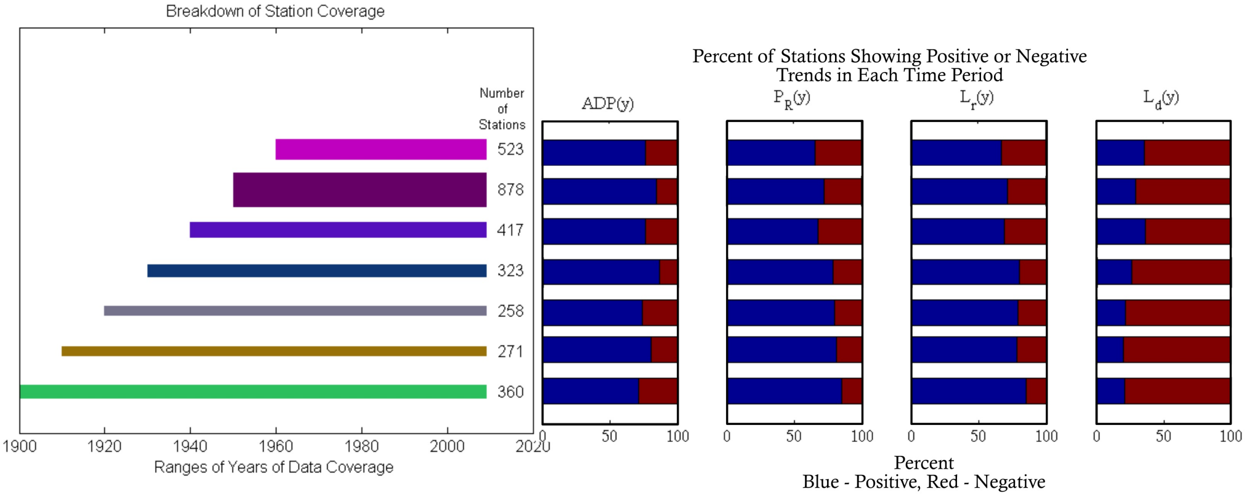

Figure S4. The left-most graph shows the breakdown of stations passing the quality control for trend analysis (3,030) separated by their start decade. The graphs to the right of this show the breakdown of positive and negative trends of the stations that showed trends in each variable. The bar beginning in 1900 represents stations whose data start in 1900 or earlier, as there are a small number of stations passing quality control criteria whose data start between 1880 and 1900.

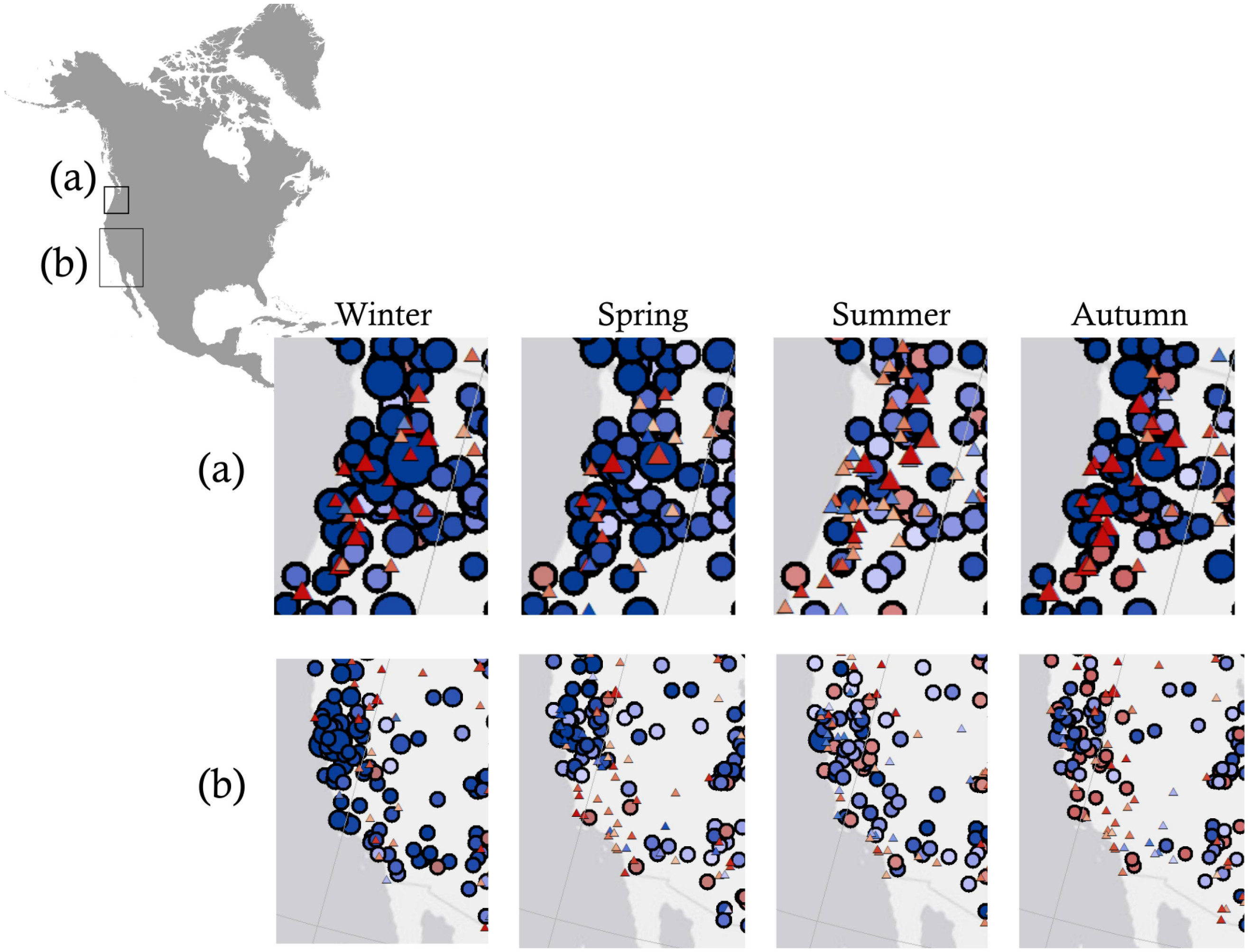

Figure S5. Seasonal analysis of non-extreme precipitation sequencing patterns for Willamette Valley (a) and the American Southwest (b). Circular symbols represent stations where $\Delta P_R(y) > 0$. Triangular symbols represent stations where $\Delta P_R(y) < 0$. The size of the symbol is proportional to the strength of the trend. The color of the symbol represents $\Delta L_r(y)$, where blue is for $\Delta L_r(y) > 0$ and red is for $\Delta L_r(y) < 0$. The more saturated the color, the larger the magnitude of the slope. Subfigures are not to scale. [Map produced with software ArcMap v. 10.4.1, http://desktop.arcgis.com/en/arcmap/.]

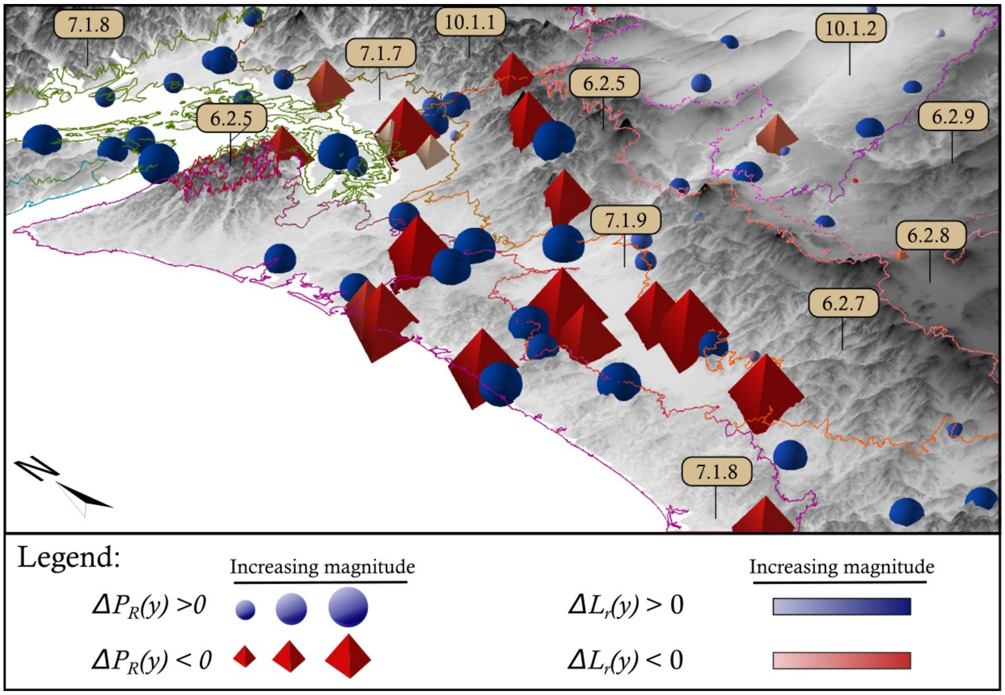

Figure S6. Detail of Pacific Northwest region. Red pyramids indicate stations with $\Delta P_R(y) < 0$ and $\Delta L_r(y) < 0$. Blue spheres indicate stations with $\Delta P_R(y) > 0$ and $\Delta L_r(y) > 0$. Willamette Valley (*III-7.1.9*) shows strongly decreasing $\Delta P_R(y)$, while surrounding areas indicate increasing $P_R(y)$. Ecoregion legend: *III-6.2.3* Northern Rockies, *III-6.2.5* North Cascades, *III-6.2.7* Cascades, *III-7.1.7* Strait of Georgia, *III-7.1.8* Coastal Range, *III-7.1.9* Willamette Valley, *III-10.1.1* Thompson-Okanagan Plateau, *III-10.1.2* Columbia Plateau. [Map produced with software ArcMap v. 10.4.1, http://desktop.arcgis.com/en/arcmap/.]
